# Unraveling the Role of N6-Methylation Modification: From Bone Biology to Osteoporosis

**DOI:** 10.7150/ijms.108763

**Published:** 2025-05-08

**Authors:** Junyi Liu, Xiang Chen, Xijie Yu

**Affiliations:** Laboratory of Endocrinology and Metabolism/Department of Endocrinology and Metabolism, Rare Disease Center, West China Hospital, Sichuan University, No. 37, Guoxue Xiang, Chengdu 610041, China

**Keywords:** N6-methyladenosine, m6A modification, bone development, bone cells, osteoporosis

## Abstract

N6-methyladenosine (m6A) is the most abundant and reversible epitranscriptomic modification in eukaryotes, playing a pivotal role in regulating various RNA metabolic processes, including splicing, nuclear export, translation and degradation. Emerging evidence indicates that m6A modification is indispensable in biological processes of bone cells such as proliferation, differentiation and apoptosis. Given its pivotal influence on osteoblastogenesis and osteoclastogenesis, m6A modification, particularly via METTL3, has attracted considerable attention in osteoporosis (OP). In this review, we probe the function of m6A modification in intramembranous and endochondral ossification. Furthermore, we summarize the regulatory role of m6A modification in various biological processes in osteoblasts, osteoclasts and osteocytes, focusing on its potential signaling pathways in osteoblast and osteoclast differentiation. Specifically, m6A modulates osteoblast differentiation predominantly through signaling pathways such as Wnt/β-catenin, PI3K/AKT, and BMP/Smad. Concurrently, it regulates osteoclast differentiation and maturation via the RANKL/RANK pathway and its downstream signaling mechanisms. We also discuss recent discoveries that m6A modification regulates OP and further explore its potential clinical value in diagnosing and treating OP. Collectively, m6A modification serves as a crucial regulatory factor in bone metabolism, and a comprehensive understanding of the molecular mechanisms of m6A modification in bone biology is expected to provide new targets for treating OP.

## 1. Introduction

Epigenetics refers to heritable changes in gene expression and function without alterations in the DNA sequence, mainly involving histone modifications, DNA methylation, and non-coding RNAs^1^. Recently, RNA methylation modifications, particularly N6-methyladenosine (m6A) methylation, have attracted widespread attention due to the popularity of high-throughput sequencing technologies^2^. m6A is the most abundant methylation modification in eukaryotic messenger RNAs (mRNAs) and also exists in non-coding RNAs such as transfer RNAs (tRNAs), ribosomal RNAs (rRNAs), and long non-coding RNAs (lncRNAs)^3^. Using sequencing technologies such as methylated RNA immunoprecipitation sequencing (MeRIP-Seq), researchers have discovered that m6A modification is significantly enriched in coding sequences (CDS), 3' untranslated regions (3' UTRs), near stop codons, and long internal exons^4^-^6^. m6A modification affects a variety of biological functions by regulating processes such as RNA transcription, splicing, translation and degradation^7^. Additionally, increasing evidence suggests that m6A modification plays essential roles in the pathogenesis of various diseases, including OP, osteoarthritis, and bone cancers, providing new therapeutic targets for these bone-related diseases^8^, ^9^.

The skeleton supports and protects the body, with the ability of dynamic remodeling. This complex process depends on the coordination among bone cells, including osteoblasts, osteoclasts, and osteocytes^10^. Osteoblasts form and mineralize bone, while osteoclasts break down and resorb bone. Osteocytes, embedded within the bone matrix, regulate the activity of osteoblasts and osteoclasts by sensing mechanical stimuli and producing endocrine factors^11^. However, aging or pathological conditions can disrupt these regulatory mechanisms, leading to abnormal bone remodeling and eventually causing osteoporosis (OP)^12^. Studies have revealed that m6A modification plays a significant role in the pathophysiology of bone^13^, ^14^. Therefore, further research clarifying how m6A modification affects bone remodeling could help identify new targets for preventing and treating OP.

In this review, we explore the effect of m6A modification on the bone development and biological processes of bone cells (osteoblasts, osteoclasts, osteocytes). Particularly, we focus on the potential pathways involved in the regulation of bone cells by m6A modification. Moreover, we discuss the implications of m6A modification for the clinical diagnosis and treatment of OP.

## 2. m6A modification: writers, erasers, readers

**Figure 1:**
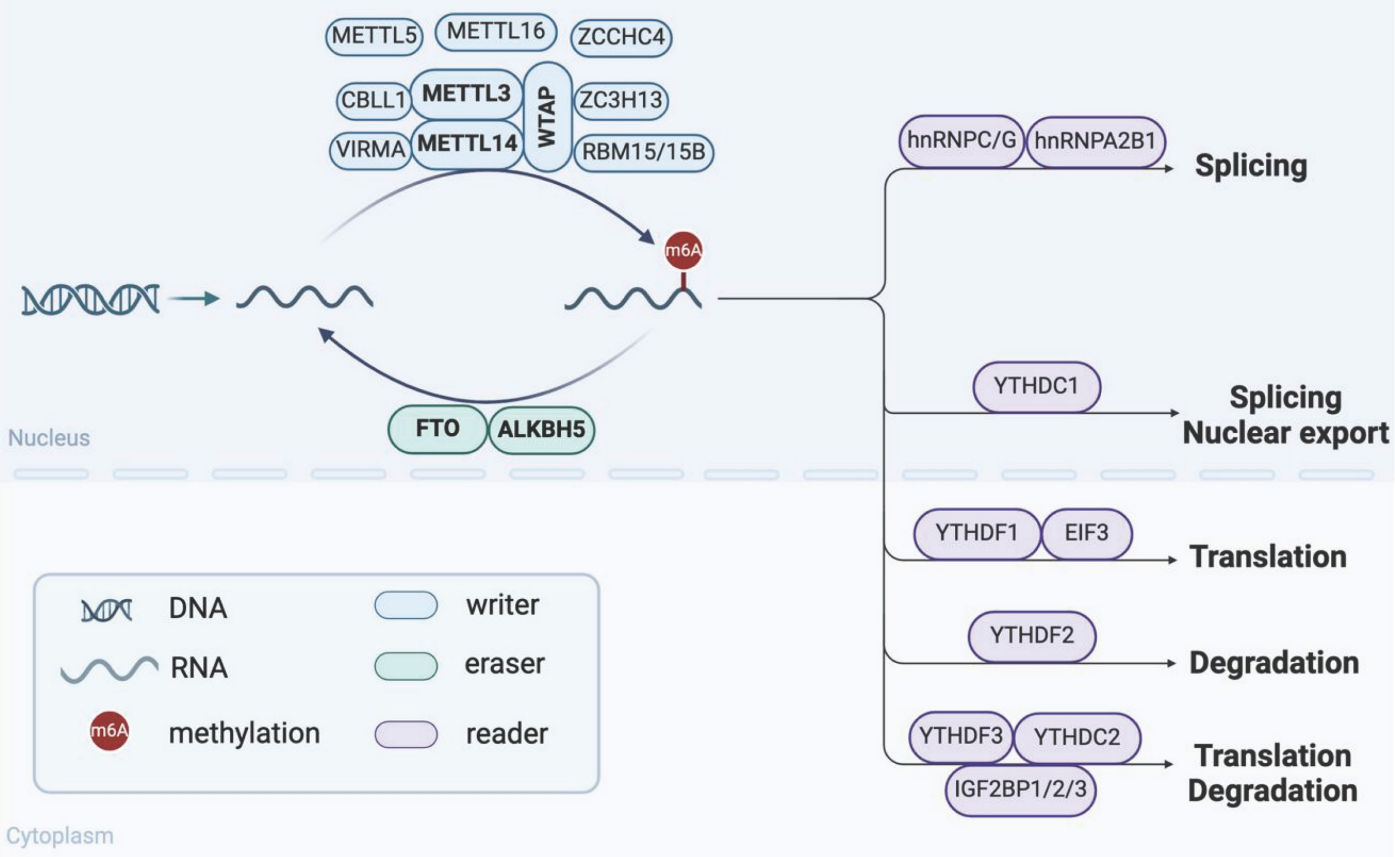
Components and regulatory mechanisms of m6A modification. The regulation of m6A modification depends on the coordination of m6A methyltransferases ("Writers"), m6A demethylases ("Erasers") and m6A-binding proteins ("Readers"). They collectively regulate various RNA biological processes, including splicing, nuclear export, translation, and degradation. (Created with BioRender.com.)

As a dynamic and reversible epigenetic mark, m6A is the most abundant post-transcriptional RNA modification in eukaryotes. m6A modification is regulated primarily by three types of regulators: m6A methyltransferases ("Writers"), m6A demethylases ("Erasers") and m6A-binding proteins ("Readers") (**Figure 1**)^15^. These regulators are responsible for adding, removing, and recognizing m6A methylation sites, respectively, thus playing crucial roles in various biological processes.

### 2.1 m6A methyltransferases (“Writers”)

The methyltransferase complex (MTC), which is essential for m6A modification, consists of core components and other regulators^15^. Methyltransferase like 3 (METTL3) is the only catalytic subunit of MTC that transfers the methyl group of S-adenosylmethionine (SAM) to adenosine^16^. The combination of methyltransferase like 14 (METTL14) and METTL3 forms a stable heterodimer complex to enhance its catalytic activity. Wilms tumor 1 associated protein (WTAP) recruits this heterodimer to mRNA specific sites to regulate m6A levels^17^. In addition, regulatory factors such as RNA-binding motif protein 15/15B (RBM15/15B), zinc finger CCCH-type containing 13 (ZC3H13), vir-like m6A methyltransferase associated (VIRMA), and Cbl proto-oncogene like 1 (CBLL1) also regulate this process^18^. Of note, Methyltransferase like 5 (METTL5), Methyltransferase like 16 (METTL16), and zinc finger CCHC-type containing 4 (ZCCHC4) function independently of the MTC. METTL5 and ZCCHC4 mediate m6A modification of 18S and 28S rRNA, respectively^19^, ^20^, while METTL16 is involved in m6A modification of U6 small nuclear RNAs (snRNAs), lncRNAs and precursor messenger RNAs (pre-mRNAs)^21^, ^22^. The cooperative action of these methyltransferases ensures the efficient operation of m6A modification.

### 2.2 m6A demethylases (“Erasers”)

The m6A demethylases mainly include fat mass and obesity-associated protein (FTO) and AlkB homolog 5 (ALKBH5), which can reverse m6A modification and participate in the dynamic regulation of m6A levels. As the first reported m6A demethylase, FTO plays an important role in regulating obesity and fat metabolism^23^. FTO-deficient osteoblasts are more susceptible to genotoxic injury under high-fat diet-induced metabolic stress^24^. ALKBH5 is recognized as the second m6A demethylase, which significantly affects mRNA export and RNA metabolism by regulating m6A levels^25^. RNA binding protein RBM33 has been confirmed to form a complex with ALKBH5, thereby regulating the demethylase activity of ALKBH5^26^.

### 2.3 m6A-binding proteins (“Readers”)

The m6A-binding protein, also known as m6A reader, affects a variety of metabolic processes of target RNA by specifically recognizing and binding to the m6A site. YT521-B homology (YTH) family proteins are essential members of the m6A readers. In the cytoplasm, YTHDF1 binds to initiation factors in the cytoplasm to promote mRNA translation, while YTHDF2 plays a dominant role in accelerating mRNA degradation^27^, ^28^. YTHDF3 is considered a collaborator of YTHDF1 and YTHDF2, playing a pivotal role in regulating mRNA dynamic balance^29^. Eukaryotic initiation factor 3 (EIF3), as a translation initiation factor, regulates translation efficiency by recognizing m6A-modified mRNA^30^. Moreover, nucleus-located YTHDC1 promotes selective splicing and nuclear export of m6A-modified mRNA by interacting with splicing factor SRSF3^31^, ^32^. YTHDC2 is closely associated with mammalian spermatogenesis, promoting translation and reducing the stability of target mRNA^33^. Besides the YTH family proteins, the insulin-like growth factor 2 mRNA binding proteins (IGF2BP)1/2/3 also maintains target mRNA stability and translational efficiency in an m6A-dependent manner^34^. It has been reported that IGF2BP3 binds to and stabilizes Beclin mRNA, which promotes the osteogenic potential of bone marrow mesenchymal stem cells (BMSCs)^35^. Heterogeneous nuclear ribonucleoproteins (hnRNPs) family also plays important roles as m6A readers. hnRNPA2B1 facilitates the processing of pri-miRNAs by interacting with DGCR8^36^. HNRNPC and HNRNPG participate in splicing pre-mRNA via m6A-mediated RNA structural switches^37^, ^38^.

Taken together, the functions of m6A regulatory factors are complex and diverse, mainly involving various biological processes such as cell proliferation, immune responses, and tumorigenesis. However, the known m6A regulatory factors are limited, and their specific mechanisms and interactions in various cell types and biological processes still require further research.

## 3. m6A modification and bone development

m6A modification regulates RNA post-transcriptional processes, influencing cell fate, tissue development and the maintenance of organ function^39^. Accumulating studies documented that m6A modification is crucial in embryonic development, with its dysregulation potentially leading to severe developmental defects or even embryonic lethality. Several pieces of evidence have identified that homozygous deletions of m6A regulatory factors, such as METTL3, METTL14 or WTAP, result in embryonic lethality in mice^40^, ^41^. The deletion of METTL3, METTL5 or METTL14 impairs the differentiation ability of mouse embryonic stem cells (mESCs), highlighting the pivotal role of m6A modification in maintaining mESCs proliferation and differentiation^42^-^44^. Furthermore, abnormal m6A modification also affects the development of multiple organs, including the liver, nerve and bone. For example, the conditional knockout of METTL3 in the embryonic liver impairs liver development and maturation^45^. The m6A reader YTHDF2 is involved in neural development and differentiation^46^. These findings offer new insights into the mechanisms underlying organ development.

Bone development is mainly achieved through intramembranous and endochondral ossification^47^. Intramembranous ossification primarily occurs in the cranium, facial bones, and clavicles. Several studies have confirmed that m6A modification is crucial in craniofacial development. METTL3-mediated m6A modification of PSEN1 regulates craniofacial development in vertebrates via the Wnt/β-catenin signaling pathway^48^. Mutations in the FTO gene result in patients with multiple malformations, which are characterized by postnatal growth retardation, craniofacial anomalies and finger deformities^49^. FTO knockout mice also exhibit a similar growth retardation phenotype, including decreased bone mineral density (BMD)^50^. In addition, normal closure of cranial sutures is crucial for intramembranous ossification of the skull^51^. METTL5 mutations have been identified as a trigger for autosomal recessive skeletal dysplasia in patients^52^. Lei et al. further showed that METTL5-deficient mice exhibit delayed suture closure and cleidocranial dysplasia^53^. Xu et al. demonstrated that cranial sutures coordinate the intramembranous ossification process of the cranial vault through METTL3. Specifically, the deletion of METTL3 in Ctsk+ calvarial stem cells resulted in delayed suture closure and skull development by inhibiting the Hedgehog signaling pathway^54^. Endochondral ossification is a process driven by the proliferation, differentiation and hypertrophy of chondrocytes, and dysfunction of chondrocytes leads to abnormal bone development^55^. In recent years, studies have found that m6A modification significantly influences the chondrocyte physiological processes and endochondral ossification. He et al. revealed that the METTL3/m6A/YTHDF1/Dmp1 axis is involved in endochondral ossification. METTL3 upregulates the expression of the target gene Dmp1 through YTHDF1-mediated m6A modification, thus promoting chondrocyte proliferation and hypertrophic differentiation^56^. METTL3 methylates circRNA3634 and upregulates the downstream target gene mitogen-activated protein kinase 1 (MAPK1), promoting the proliferation and differentiation of antler chondrocytes^57^. Moreover, METTL3 is essential for maintaining endochondral ossification of condylar cartilage. METTL3 deficiency in Acan+ chondrocytes leads to abnormal mandibular condylar chondrogenesis by upregulating the expression of Lats1, a key molecule of the Hippo/Yap1 pathway^58^.

Bone development is regulated by key transcription factors such as Runx2, Osterix, SOX9, and ATF4^59^. Numerous studies have found that m6A modification regulates these transcription factors via m6A regulatory factors, affecting osteogenesis and chondrogenesis commitment. Specifically, METTL3, METTL14, WTAP, ALKBH5, YTHDF1 and YTHDF3 promote osteoblast differentiation by directly or indirectly upregulating Runx2 expression^60^-^65^. Additionally, m6A modification mediated by METTL3 and YTHDF3 also enhances the expression of Osterix, which cooperates with Runx2 to regulate the osteogenic process^63^, ^66^. However, FTO and YTHDC2 negatively regulate Runx2 expression, inhibiting osteogenic differentiation^67^, ^68^. SOX9 is a key transcription factor mediating chondrogenic differentiation and bone development. METTL3 has been proven to target the 3'UTR of SOX9 mRNA and regulate SOX9 translation during cartilage differentiation^69^. Furthermore, another study reported that tension-stimulated METTL3 inhibited extracellular matrix synthesis in endplate chondrocytes by mediating SOX9 m6A modification and inducing its degradation^70^.

Collectively, m6A modification regulates intramembranous and endochondral ossification and maintains normal bone development by modulating relevant transcription factors and signaling pathways.

## 4. The regulatory effect of m6A modification in bone cells

**Figure 2:**
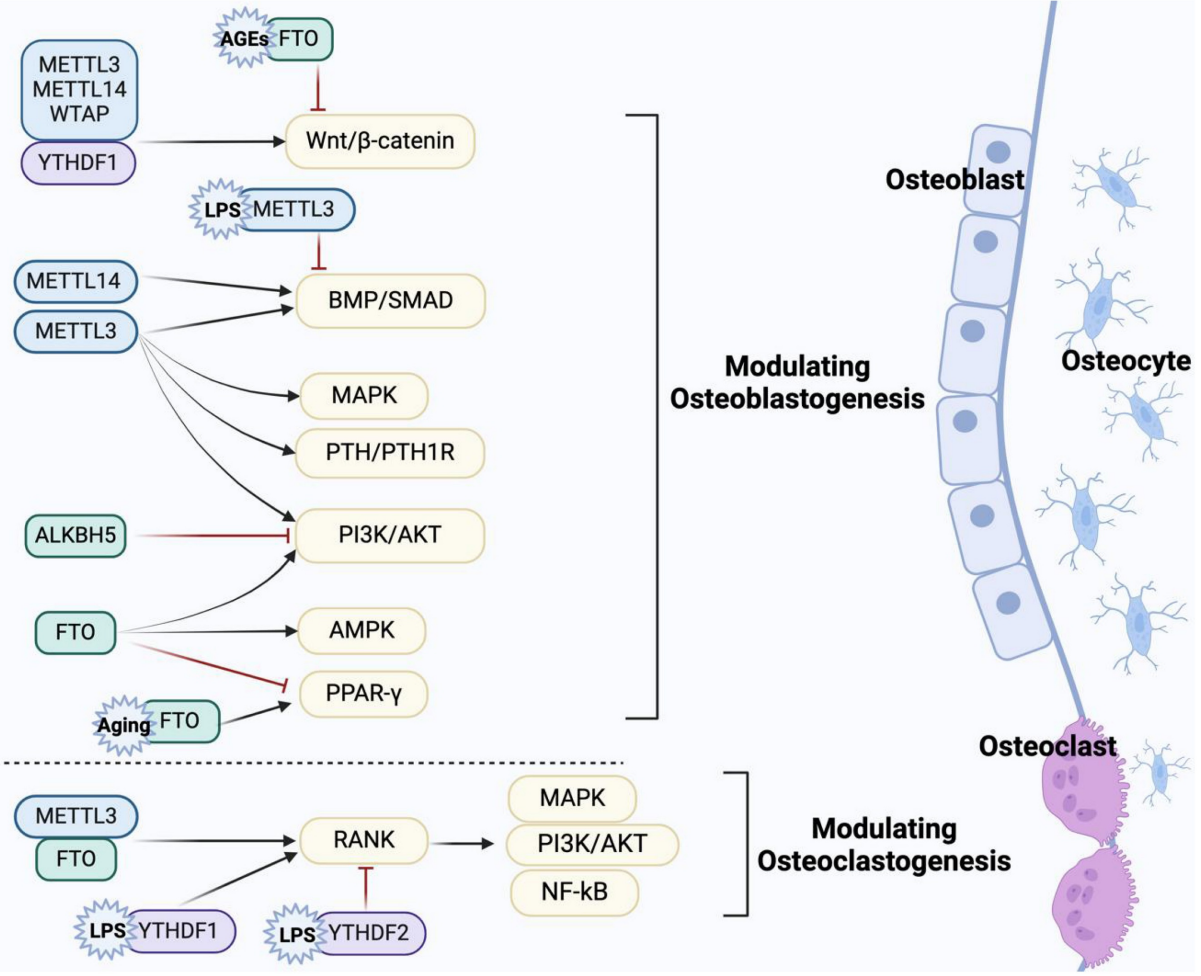
The pathways involved in the regulation of osteoblast and osteoclast differentiation by m6A modification. m6A regulators are involved in osteoblast differentiation via Wnt/β-catenin, BMP/Smad, PI3K/AKT, and other potential signaling pathways, while regulating osteoclast differentiation via RANKL/RANK and its downstream pathways. In addition, under pathological conditions such as aging, AGE and LPS, m6A regulators also affect bone-resorbing osteoclasts and bone-forming osteoblasts through relevant pathways. (Created with BioRender.com.)

The coordination of bone cells (osteoblasts, osteoclasts, osteocytes) is responsible for the dynamic remodeling of bone. Existing evidence suggests that m6A modification not only regulates the proliferation and apoptosis of bone cells (**Table 1**), but also participates in the differentiation of osteoblasts and osteoclasts via multiple signaling pathways (**Figure 2**). Hence, clarifying the precise regulatory mechanism of m6A modification on the biological processes of bone cells contributes to a better understanding of bone metabolism and bone-related diseases.

### 4.1 The regulatory effect of m6A modification on osteoblasts

Osteoblasts undergo multiple stages during bone formation, including proliferation, differentiation, mineralization and apoptosis. m6A modification is involved in maintaining the dynamic balance of bone by regulating these processes. It was reported that METTL3 knockdown mediates osteoblast proliferation, differentiation and apoptosis by activating the endoplasmic reticulum stress signaling pathway^71^. FTO targets the HSPA1a/NF-κB signaling axis, inhibiting genotoxicity-induced osteoblast apoptosis^24^. Osteoblast progenitor cells differentiate into mature osteoblasts through four stages: proliferation, differentiation, matrix synthesis, and mineralization. During this process, osteoblasts synthesize and secrete the bone matrix, facilitating mineral deposition to form new bone tissue^72^. Osteoblast differentiation is critical for proper bone formation. Key transcription factors, such as Runx2 and Osterix, play central roles in this process, with their functions driven by signaling pathways such as Wnt/β-catenin and bone morphogenic protein (BMP)^73^. Proper osteoblast differentiation ensures bone matrix synthesis and mineralization, whereas its dysfunction may lead to decreased bone mass or skeletal developmental defects^73^, ^74^. Of note, m6A modification plays a crucial role in the differentiation of osteoblasts. Herein, we systematically summarize the osteogenic differentiation pathways related to m6A modification.

#### 4.1.1 m6A regulates osteogenic differentiation by Wnt/β-catenin pathway

It is well known that the Wnt signaling pathway plays a pivotal role in osteoblast-mediated bone formation, and β-catenin is an essential regulatory factor in the canonical Wnt pathway^75^. Accumulating evidence suggests that activation of the Wnt/β-catenin signaling pathway promotes the proliferation, differentiation and maintenance of osteoblasts^76^. Recent studies have emphasized the potential role of m6A modification in osteogenic differentiation by regulating the Wnt/β-catenin pathway. Wu et al. demonstrated that METTL3 overexpression partially rescues the osteogenic potential of BMSCs in OP rats by activating the Wnt signaling pathway^77^. METTL3 can also activate the Wnt/β-catenin/c-Myc signaling pathway in a YTHDF2-dependent manner in the inflammation induced by lipopolysaccharides (LPS), contributing to the biological behaviors of osteoblasts^78^. METTL14 upregulates TCF1 expression by m6A modification. As a key transcription factor in the Wnt/β-catenin pathway, TCF1 enhances Runx2 expression to alleviate OP, suggesting that METTL14 may regulate the Wnt/β-catenin pathway^65^. WTAP-mediated m6A modification of miR-181a and miR-181c contributes to the osteogenesis of BMSCs by suppressing the expression of the target secretory frizzled-related protein 1 (SFRP1)^79^. SFRP1 has been confirmed as a negative regulator of the Wnt pathway involved in the lineage differentiation of BMSCs^80^, ^81^. This suggests that WTAP may indirectly modulate the Wnt signaling pathway, thereby playing a significant role in bone homeostasis. Furthermore, m6A erasers and readers also engage in the osteogenic differentiation related to the Wnt pathway. Gao et al. showed that YTHDF1 activates Wnt/β-catenin signaling through autophagy, thereby promoting osteogenic differentiation and proliferation^82^. FTO methylates sclerostin (SOST) transcripts, thereby mediating osteogenic impairment induced by advanced glycation end products (AGEs) via the Wnt signaling pathway^83^.

#### 4.1.2 m6A regulates osteogenic differentiation by BMP/Smad pathway

The BMP signaling pathway plays a critical biological role in bone development and fracture healing^84^. As one of the important subtypes of the BMP family, BMP2 binds to its receptor BMPR to activate downstream Smad1/5/8 phosphorylation, which mediates osteoblast differentiation by up-regulating the expression of osteogenic-specific genes such as Runx2, Osterix^85^, ^86^. Liu et al. found that BMP2 is a downstream target of METTL3, and m6A activity is inhibited by piRNA-36741. piRNA-36741 can enhance the expression of BMP2 by binding to METTL3, thereby promoting the osteogenic differentiation of BMSCs^87^. Meanwhile, METTL3 positively correlates with receptor BMPR1B expression. METTL3 promotes competitive binding of LINC00657 to miR-144-3p and targets the LINC00657/miR-144-3p/BMPR1B axis to initiate osteoblast differentiation and bone formation^88^. In addition, the low METTL14 can downregulate the expression of Smad1, Smad5 and Smad8 in BMSCs, impairing osteogenic differentiation by inhibiting the m6A modification of Smad1^89^. Smad7 and Smurf1 are inhibitors of the BMP signaling pathway. Smad7 recruits E3 ubiquitin ligases such as Smurf1, leading to ubiquitinated degradation of the phosphorylated receptor^90^, ^91^. METTL3 knockdown promotes the stability of Smad7 and Smurf1 in MC3T3-E1 cells, thereby negatively regulating Smad-dependent signaling and osteogenic differentiation under inflammatory conditions^92^. These studies revealed that m6A modification in osteogenic differentiation is related to BMP/Smad pathways.

#### 4.1.3 m6A regulates osteogenic differentiation by PI3K/AKT pathway

The phosphoinositide 3-kinase/AKT (PI3K/AKT) pathway is a key signaling pathway that regulates cell proliferation, differentiation and survival^93^. PI3K phosphorylates PIP2 to produce PIP3, which acts as a second messenger to recruit and activate the downstream serine/threonine-protein kinase AKT, mediating various cellular physiological and pathological processes^94^, ^95^. In the skeletal system, activation of the PI3K/AKT pathway is involved in osteoblast proliferation, differentiation and apoptosis, thereby facilitating the progression of OP^96^, ^97^. For example, an *in vitro* study suggested that lower levels of METTL3 downregulate the PI3K/AKT pathway, triggering impaired osteogenic differentiation in BMSCs^98^. Li et al. found that mice with specific knockout of ALKBH5 in BMSCs have significantly higher bone mass than littermate controls. They revealed that ALKBH5 negatively regulates osteogenic differentiation in BMSCs by targeting protein arginine methyltransferase 6 (PRMT6), which inactivates the downstream PI3K/AKT pathway^99^. Furthermore, overexpressed FTO in ovariectomy (OVX) mice is inhibited by miR-22-3p derived from BMSCs, which promotes osteogenic differentiation through the MYC/PI3K/AKT pathway^100^. Besides, the intact insulin-like growth factor (IGF)-induced PI3K/AKT signaling cascade is crucial for osteoblast differentiation and bone development^101^. As a key component of the IGF signaling pathway, IGF-2 mediates signal transduction through the IGF receptor, subsequently activating the PI3K/AKT signaling pathway^101^. It has been reported recently that the glutamine-αKG axis suppresses the translation efficiency of IGF2 in an m6A modification-dependent manner, thereby modulating the osteo/odontogenic differentiation of mesenchymal adult stem cells (m-ASCs)^102^. These results indicate that m6A plays an important role in osteogenic differentiation by the PI3K/AKT signaling pathway.

#### 4.1.4 m6A regulates osteogenic differentiation by MAPK pathway

The MAPK pathway is crucial for receiving extracellular stimuli and conveying these signals into the intracellular environment^103^. ERKs, JNKs and p38 constitute the classical MAPK signaling pathway, collaboratively engaging in cellular responses to diverse external stimuli via distinct functions and regulatory mechanisms^104^, ^105^. In MC3T3-E1 cells, METTL3 depletion significantly increases the phosphorylation levels of ERK, JNK and p38 within the MAPK signaling pathway, thereby activating the inflammatory response of osteoblasts^92^. Intriguingly, Song et al. reported that METTL3-mediated m6A modification can increase the phosphorylation levels of p38, JNK and ERK and subsequently activate the MAPK signaling pathway, participating in the osteogenic differentiation of human adipose derived stem cells (ASCs)^106^. Lin et al. revealed that overexpression of METTL3 involves in osteoblast ferroptosis by activating the ASK1/p38 signaling pathway under high glucose and high fat conditions^107^. In contrast, they found inhibition of p38 phosphorylation significantly alleviated osteoblast dysfunction^107^. These findings suggest that m6A modification may be involved in the pathological processes of osteoblasts and the development of bone diseases through abnormal dysregulation of ERKs, JNKs and p38. This provides evidence to support further exploration of the precise mechanisms by which the m6A and MAPK pathways are involved in osteoblast differentiation, as well as the identification of novel therapeutic targets.

#### 4.1.5 m6A regulates osteogenic differentiation by other potential pathways

Notably, m6A modification is also involved in osteogenic differentiation by regulating other pathways such as adenosine monophosphate-activated protein kinase (AMPK) and parathyroid hormone/parathyroid hormone 1 receptor (PTH/PTH1R). An *in vitro* study confirmed that the m6A demethylase FTO promoted the osteogenic differentiation of C3H10T1/2 cells by activating the AMPK signaling pathway and inducing mild endoplasmic reticulum stress through a BMP2-induced FTO/p-AMPK positive feedback loop^108^. Chen et al. reported that FTO overexpression during aging inhibits osteoblast differentiation and OP progress by increasing the expression of peroxisome proliferator-activated receptor γ (PPARγ)^109^. Nevertheless, another study showed that FTO could target the 3' UTR of PPARγ mRNA and negatively regulate the PPARγ signaling pathway, thereby increasing the expression of osteogenic marker genes such as alkaline phosphatase (ALP) and osteopontin (OPN)^110^. The inconsistency requires further studies to explore the interaction between FTO and PPARγ signaling pathway in OP. In osteoblasts and osteocytes, the PTH signaling pathway is essential for regulating bone metabolism and alleviating age-related bone loss^111^, ^112^. METTL3 deficiency in BMSCs has been shown to inhibit osteogenesis in mice. Mechanistically, the deficiency of METTL3 can block PTH/PTH1R signaling by reducing the translation efficiency of PTH1R, ultimately leading to the differentiation of BMSCs into adipocytes instead of osteoblasts^113^. These findings provide new mechanistic insights into the role of m6A modification in osteogenic differentiation.

### 4.2 The regulatory effect of m6A modification on osteoclasts

Osteoclasts are critical cells in bone resorption and are responsible for bone development, growth, and remodeling^114^. Notably, mononuclear osteoclast precursors have limited resorptive capacity, which significantly increases only after they undergo a series of complex processes, including migration, recognition, intercellular adhesion, and membrane fusion, to differentiate into multinucleated mature osteoclasts^114^. This differentiation is primarily regulated by the RANK/RANKL/OPG signaling pathway. Mature osteoclasts establish a sealing zone and secrete acidic substances and proteases to degrade the bone matrix, thus completing the resorption process^115^. A recent study has demonstrated that FTO is mainly involved in the proliferation and apoptosis of osteoclast precursors by upregulating the expression of CDK2 and cyclin A2 while inhibiting the expression of DNA damage-related proteins^116^. However, FTO inhibitor treatment significantly reduces the number of multinucleated osteoclasts and bone resorption ability 116. Li et al. found that METTL3 inhibits osteoclast apoptosis via iNOS/NO-mediated mitochondrial dysfunction under inflammatory conditions^117^. Intriguingly, an *in vitro* study showed that knockdown of METTL3 and YTHDF2 upregulated the stability of Atp6v0d2 mRNA, leading to the formation of multinucleated osteoclasts, without affecting the proliferation of osteoclast precursor^118^. These findings suggest that m6A modification is involved in the regulation of osteoclast proliferation, apoptosis and activity. Migration of osteoclasts during bone resorption is critical for the progress of OP. Recently, an *in vitro* study reported that follicle-stimulating hormone (FSH) enhances the m6A activity of METTL3 through cyclic-AMP response element-binding protein (CREB), which increases cathepsin K (CTSK) mRNA stability and subsequently promotes osteoclast migration^119^. This result provides a new target for utilizing m6A in the treatment of postmenopausal OP. Moreover, m6A modification is closely associated with osteoclast differentiation under different pathological conditions. Early growth response 1 (EGR1) was shown to promote METTL3 transcription and enhance the chitinase 3-like protein 1 (CHI3L1) m6A methylation, thus stimulating osteoclast differentiation in OVX mice^120^. Shen et al. reported that toll-like receptor 4 (TLR4) promotes high glucose and high fat-induced osteoclast differentiation by inhibiting FTO-mediated m6A modification, suggesting that m6A modification participated in developing diabetic bone loss^121^. Specifically, METTL14 inhibited bone-resorbing osteoclasts by binding to the methylation functional site of nuclear factor of activated T-cells cytoplasmic 1 (NFATc1)^122^. In addition, METTL14 enhances glutathione peroxidase 4 (GPX4) mRNA stability under the influence of human antigen R (HuR), thereby inhibiting osteoclast formation and bone resorption^123^. Similarly, YTHDC1 enhanced protein tyrosine phosphatase non-receptor type 6 (PTPN6) expression in an m6A-HuR-dependent manner to inhibit osteoclast differentiation and OP progression^124^. Liu et al. reported that knockdown of WTAP significantly increases the expression of osteoclast-related genes^125^, indicating that WTAP-mediated m6A modification serves an inhibitory role in osteoclast differentiation.

Receptor activator of NF-κB ligand (RANKL) is a critical factor in osteoclastogenesis. Upon binding to its receptor activator of NF-κB (RANK) on osteoclast precursors, RANKL recruits various adaptor molecules, particularly tumor necrosis factor receptor-associated factor 6 (TRAF6). This recruitment activates downstream signaling pathways, including NF-κB, MAPK and PI3K/AKT, thus promoting the maturation of osteoclasts and facilitating bone resorption^126^, ^127^. He et al. found that METTL3 knockdown prevents the nuclear export of TRAF6 mRNA, leading to the inactivation of downstream signaling pathways of RANKL/RANK and inhibiting the differentiation and formation of osteoclasts^118^. They also demonstrated that YTHDF1 is involved in inflammatory osteoclastogenesis. YTHDF1 depletion can inactivate the NF-κB, MAPK and PI3K/AKT pathways by affecting the stability of the RANK mRNA and ultimately inhibits LPS-induced osteoclastogenesis^128^. In contrast, an *in vitro* study reported that the YTHDF2 knockdown in osteoclast precursor promotes osteoclastogenesis under inflammatory stimuli, primarily by activating the NF-κB and MAPK signaling pathways^129^. Of note, the NF-κB pathway is one of the most important pathways mediating osteoclast differentiation following the binding of RANK and RANKL^130^. FTO activates the NF-κB signaling pathway, promoting osteoclast differentiation and bone resorption. However, treatment with NF-κB inhibitor attenuates the positive regulatory effect of FTO^131^. These studies provide evidence that m6A modification is involved in regulating osteoclast differentiation via the RANKL/RANK and its downstream pathways. Additionally, Osteoprotegerin (OPG) acts as a decoy receptor for RANKL, inhibiting osteoclastogenesis by preventing the binding of RANKL to RANK^115^. Thus, the RANKL/OPG ratio is essential for regulating bone resorption. Studies have shown that m6A modification influences this ratio. Specifically, overexpression of METTL14 decreases RANKL levels while increasing OPG expression in osteoblasts, thereby enhancing osteoclast differentiation^65^. IGF2BP2 has also been shown to affect the RANKL/OPG ratio under inflammatory conditions^132^.

### 4.3 The regulatory effect of m6A modification on osteocytes

Osteocytes, the most abundant cell type in bone tissue, possess unique dendritic structures that interconnect through a network of canaliculi^133^. These structures enable osteocytes to sense and respond to mechanical loads to maintain the dynamic balance of bone mass. Studies have indicated that epigenetic modification, particularly histone modifications, plays a significant role in the development and function of osteocytes. For example, Datta and his team established the initial link between mechanical loading and epigenetic changes in bone. They found that mechanical loading epigenetically regulates the expression of the zinc finger of the cerebellum 1 (ZIC1), which influences the transcriptional activity of osteocyte markers^134^. Stegen et al. revealed that the absence of the oxygen sensor prolyl hydroxylase 2 (PHD2) in osteocytes downregulates SOST expression through SIRT1-mediated epigenetic modification, thereby activating the Wnt/β-catenin signaling pathway to increase bone mass^135^. Moreover, the histone demethylase Utx in osteocytes positively regulates their differentiation and maturation by removing H3K27me3-mediated histone modification^136^. Recently, m6A modification has been shown to regulate osteocyte structure and function. For example, Xu et al. demonstrated that the specific knockout of METTL3 in non-osteoclastic Ctsk+ lineage cells resulted in a reduced number of osteocytes with impaired development, characterized by fewer canaliculi^54^. Nevertheless, the specific regulatory mechanism of m6A modification in osteocytes remains largely unknown. As such, further research is required to clarify the complex relationship between m6A modification and osteocytes, which could pave the way for novel therapeutic strategies targeting OP and other skeletal diseases.

### 4.4 The regulatory effect of m6A modification on other cells

Besides osteoblasts and osteoclasts, other cell types, such as vascular endothelial cells and immune cells, also play crucial roles in bone remodeling. Macrophages, as a special cell type within bone tissue, significantly influence immune regulation and inflammatory responses through their polarization^137^. Under inflammatory conditions, downregulation of WTAP can promote macrophage polarization towards the M2 phenotype, thereby enhancing osteogenic differentiation of BMSCs^138^. METTL3 regulates macrophage polarization by mediating m6A modification of HDAC5 and further participates in bone repair processes^139^. These evidences suggest that m6A modification plays a critical role in coordinating macrophage-mediated bone immune responses. Furthermore, pyroptosis contributes to the development of OP by modulating the inflammatory immune microenvironment^140^. Tang et al. reported that overexpression of METTL14 inhibits macrophage-osteoclast differentiation and macrophage pyroptosis. Mechanistically, METTL14 targeting the METTL14/HOXA5/WNK1 axis to suppress NLRP3-dependent pyroptosis, thereby alleviating OP^141^. Yang et al. applied a diabetes-associated periodontitis model to demonstrate that METTL3 induces macrophage pyroptosis by methylating NLRP3 mRNA, thereby accelerating alveolar bone loss^142^.

Angiogenesis, which is closely linked to osteogenesis, is essential for maintaining bone homeostasis^143^. The vascular endothelial growth factor A (VEGFA) signaling pathway mediated by endothelial cells plays a key role in bone vascularization^144^. An *in vitro* study indicated that METTL3 modulates the expression of VEGFA and its splice variants during osteogenic differentiation of BMSCs, highlighting a potential role for m6A in regulating angiogenesis through endothelial cell-associated genes^98^. Jiang et al. further revealed that METTL3 is a critical regulator of angiogenesis in endothelial progenitor cells (EPCs). METTL3 participates in the proliferation, migration, and tube formation of EPCs through the PI3K/AKT signaling pathway, ultimately promoting distraction osteogenesis-related ossification^145^. Studies should further investigate the specific role of m6A modification in bone remodeling across these cell types, particularly its interactions with bone cells.

## 5. m6A modification and osteoporosis

### 5.1 Evidence from animal and cellular studies

OP is a metabolic bone disease characterized by reduced bone mass and deterioration of bone microarchitecture^146^. With the global aging population, the incidence of OP is rising, significantly affecting the quality of life in patients^146^, ^147^. The pathogenesis of OP is closely related to the dysregulation of bone metabolism caused by the osteoblast-osteoclast uncoupling and imbalance of BMSCs differentiation^148^. Researchers suggest that m6A modification plays a vital role in OP. Based on extensive studies on animal models and cell cultures, Liang et al. summarized the regulatory mechanisms of m6A in osteoblasts, osteoclasts and BMSCs, emphasizing its crucial role in OP^149^. Specifically, m6A modification regulates bone homeostasis through various mechanisms. It influences osteoclast differentiation to modulate bone resorption, controls osteoblast activity for new bone formation, and directs BMSCs differentiation toward osteogenesis while inhibiting adipogenesis. Key m6A regulators, such as METTL3 and METTL14, orchestrate these processes, positioning them as promising targets for OP treatment^149^. Recent studies further reveal new insights into m6A and senile OP. Wang et al. discovered that the expression of METTL3 was significantly decreased in the senile OP mouse model. METTL3 partially reversed the aging of osteoblasts and subsequent age-related bone loss by increasing the stability of Hspa1a mRNA^150^. Cell culture study also confirmed that downregulation of METTL3 promotes the degradation of LINC01013, thereby inhibiting the differentiation of pre-osteoblasts during aging^151^. However, another study showed that METTL3 facilitates osteoblast senescence via the METTL3/IGF2BP2/Slc1a5 axis, thereby exacerbating age-related bone loss^152^. Additionally, an *in vitro* study demonstrated that ALKBH5 regulates cellular senescence and osteogenic differentiation in age-related OP by modulating the m6A modification of voltage-dependent anion channel 3 (VDAC3)^153^. The discrepancies observed in these researches may result from differing experimental conditions, emphasizing the necessity for further exploration of how m6A affects OP in different pathological conditions.

### 5.2 Evidence from human studies

In recent years, m6A epigenetic modification has emerged as a burgeoning field, providing new perspectives for understanding the pathophysiology of bone diseases^13^. Several clinical studies have provided evidence supporting a close association between m6A regulatory factors and metrics indicative of bone health, particularly BMD. Deng et al. observed low levels of METTL14 in the serum of postmenopausal women with OP, which were positively correlated with BMD^123^. Similarly, Serum YTHDC1 levels were positively correlate with lumbar spine BMD in OP patients^124^. Notably, single nucleotide polymorphisms (SNPs) have been associated with a variety of disease susceptibilities, and m6A-related SNPs can also influence the progression of OP. Han and his team identified a large number of m6A-SNPs were associated with BMD through genome-wide association studies (GWAS)^154^, ^155^. Meanwhile, several follow-up studies confirmed these findings, revealing significant associations between FTO SNPs and hip BMD as well as osteoporotic fracture risk^156^-^158^. Additionally, numerous studies have shown that osteoporotic bone tissue exhibits significantly lower levels of m6A compared to normal healthy individuals, primarily because of decreased expression of m6A methyltransferases METTL3^66^, ^88^, ^150^, ^159^, METTL14^65^, ^89^ and WTAP^79^, ^125^, as well as increased expression of the m6A demethylase FTO^67^, ^110^. However, evidence provided by Dong et al. indicates that the levels of m6A and METTL14 are upregulated in the bone tissue of patients with OP^160^. Therefore, future clinical studies with larger sample sizes are necessary to accurately reveal the specific relationship between m6A regulators and OP.

### 5.3 Clinical value of m6A in osteoporosis

Due to the lack of reliable biomarkers for the prediction and diagnosis of OP, the discovery of novel biomarkers using histological techniques, especially the epitranscriptome, could be useful for early screening and clinical decision-making in OP^161^. m6A modification as an important RNA epigenetic modification shows potential value in the prediction and diagnosis of OP. For example, a diagnostic model for OP constructed based on the Gene Expression Omnibus (GEO) dataset identified four m6A regulator factors: METTL16, CBLL1, YTHDF2, and FTO. Notably, CBLL1 and YTHDF2 were recognized as protective factors, whereas METTL16 and FTO were classified as risk factors^162^, ^163^. Zhang et al. identified seven m6A regulators, including FTO, FMR1, YTHDC2, HNRNPC, RBM15, RBM15B and WTAP in human peripheral blood mononuclear cells (PBMCs) by bioinformatics analysis, which may serve as potential biomarkers for diagnosis of postmenopausal OP^164^. These findings provide new insights into the exploration of biomarkers associated with OP. However, further validation of their clinical reliability and practicality is necessary.

In terms of treatment, although anti-osteoporosis medications offer significant therapeutic benefits for patients, their potential risks and side effects may limit long-term use^165^, ^166^. Therefore, developing novel medications with fewer side effects for treating OP remains a crucial strategy. As mentioned previously, m6A regulates the proliferation, differentiation and maturation of osteoblasts and osteoclasts, which provides strong evidence for targeting m6A as a therapeutic approach in OP. Recently, several studies have identified potential drugs for treating OP by modulating m6A (**Table 2**). Jin et al. revealed that icariin enhances the m6A levels of P4HB through METTL14, thereby promoting the osteogenic differentiation of BMSCs^167^. Chinese Ecliptae herba extract and its component wedelolactone can target METTL3, activating various pathways such as HIF-1α, PI3K, and Hippo to alleviate OP^168^. Xianling Gubao Capsule and Qianggu Decoction also rescue the decreased osteogenic potential of BMSCs in OP via METTL3-mediated m6A modification^169^, ^170^. Intriguingly, Wei et al. identified Specnuezhenide as a promising candidate for OP treatment through single-cell sequencing, showing its ability to enhance angiogenesis and osteogenesis in LepR+ BMSCs by activating METTL3^171^. In addition, several clinical studies have confirmed the positive effects of these drugs in the treatment of OP. For example, meta-analysis based on randomized clinical trials revealed that Xianling Gubao capsules have the potential to improve quality of life and alleviate bone pain in the treatment of primary OP^172^. A 24-month randomized controlled clinical trial provided evidence that Epimedium-derived compounds, primarily consisting of icariin, significantly attenuated bone loss in postmenopausal women^173^. Yong et al. further demonstrated that the purified Epimedium extract exhibited a favorable safety profile following continuous administration for 6 weeks in postmenopausal women^174^. These results suggest that targeting m6A shows excellent potential in the treatment of OP. Nevertheless, therapeutic agents targeting m6A remain in the early phases of clinical application. Although several drugs have shown promising efficacy in preclinical studies, large-scale clinical trials are still scarce. In the future, as more clinical studies targeting m6A progress, further development of novel m6A-based targeted drugs is expected to provide more effective and personalized clinical benefits to patients with OP.

Furthermore, numerous studies have confirmed that m6A plays an essential role in the prognosis of cancers (i.e., liver cancer, breast cancer and osteosarcoma)^175^-^177^, which indicates that m6A regulatory factors may serve as potential biomarkers in evaluating the prognosis of OP. Taken together, m6A methylation modification has a broad application in the prediction, diagnosis, treatment, and prognostic management of OP.

## 6. Conclusion and future perspectives

In summary, m6A modification is a key contributor to the biological processes of bone cells, particularly by regulating various signaling pathways that affect their differentiation and activity. Specifically, m6A regulatory factors modulate osteoblast differentiation and mineralization through signaling pathways such as Wnt/β-catenin, PI3K/AKT, and BMP/Smad. Meanwhile, they regulate osteoclast differentiation and bone resorption via the RANKL/RANK pathway and its downstream signaling mechanisms. Moreover, the number and development of osteocytes are also influenced by m6A modification. Notably, abnormal m6A modification may impair the bone remodeling, contributing to the progression of metabolic bone diseases such as OP. However, the specific mechanisms of m6A modification in bone pathophysiology remain largely unknown.

Therefore, future research should prioritize the following aspects: First, the onset of OP may involve the collaborative effects of multiple m6A regulatory proteins, requiring further exploration of novel m6A regulators and their interactions in bone pathophysiology. Second, it is essential to clarify the specific roles of m6A modification in different bone cells, especially osteoclasts and osteocytes. Creating specific knockout mouse models lacking m6A regulatory factors will be essential for advancing this research. Finally, given the complex relationship between m6A modification and bone remodeling, future efforts should develop novel anti-osteoporotic drugs targeting m6A and translate these findings into effective clinical diagnostic and therapeutic tools for OP.

## Figures and Tables

**Table 1:** The role of m6A regulatory factors in the biological processes of bone cells

m6A regulators	Expression	Target genes	Function	References
**Osteoblast**				
METTL3	↓	Grp78	Promoting osteoblast apoptosis and inhibiting proliferation and differentiation	71
FTO	↑	Hspa1a/NF-κB	Inhibiting osteoblast genotoxic-induced apoptosis	24
**Osteoclast**				
FTO	↑	CDK2, Cyclin A2 and DNA damage-related proteins	Promoting osteoclast proliferation and inhibiting apoptosis	116
METTL3	↓	Nos2	Inhibiting osteoclast differentiation and promoting apoptosis	117
METTL3/YTHDF2	↓	Atp6v0d2	Inhibiting osteoclast differentiation and activity	118
METTL3	↑	CTSK	Promoting osteoclast migration	119
Osteocyte				
METTL3	↓	-	Inhibiting the number and development of osteocyte	54

**Table 2:** Potential anti-osteoporosis drugs targeting m6A modification.

Potential drugs	Target	Mechanism	Pharmacological function	References
Icariin	METTL14	METTL14/P4HB	Promoting osteogenesis	167
Chinese Ecliptae herba	METTL3	HIF-1α, PI3K/AKT and Hippo pathway	Promoting osteogenesis	168
Qianggu Decoction	METTL3	METTL3/Runx2	Promoting osteogenesis	169
Xianling Gubao Capsule	METTL3	-	Promoting osteogenesis	170
Specnuezhenide	METTL3	METTL3/Runx2 METTL3/SLIT3	Promoting LepR+ BMSCs-dependent osteogenesis and angiogenesis	171

## Abbreviations

m6A: N6-methyladenosine
OP: Osteoporosis
mRNAs: messenger RNAs
tRNAs: transfer RNAs
rRNAs: ribosomal RNAs
lncRNAs: long non-coding RNAs
MeRIP-Seq: Methylated RNA immunoprecipitation sequencing
CDS: Coding sequences
3' UTRs: 3' untranslated regions
MTC: Methyltransferase complex
METTL3: Methyltransferase like 3
SAM: S-adenosylmethionine
METTL14: Methyltransferase like 14
WTAP: Wilms tumor 1 associated protein
RBM15/15B: RNA-binding motif protein 15/15B
ZC3H13: Zinc finger CCCH-type containing 13
VIRMA: Vir-like m6A methyltransferase associated
CBLL1: Cbl proto-oncogene like 1
METTL5: Methyltransferase like 5
METTL16: Methyltransferase like 16
ZCCHC4: Zinc finger CCHC-type containing 4
snRNAs: Small nuclear RNAs
pre-mRNAs: precursor messenger RNAs
FTO: Fat mass and obesity-associated protein
ALKBH5: AlkB homolog 5
YTH: YT521-B homology
EIF3: Eukaryotic initiation factor 3
IGF2BP: Insulin-like growth factor 2 mRNA binding proteins
BMSCs: Bone marrow mesenchymal stem cells
hnRNPs: Heterogeneous nuclear ribonucleoproteins
mESCs: Mouse embryonic stem cells
BMD: Bone mineral density
MAPK: Mitogen-activated protein kinase
BMP: Bone morphogenic protein
LPS: Lipopolysaccharides
SFRP1: Secretory frizzled-related protein 1
SOST: Sclerostin
AGEs: Advanced glycation end products
PI3K: Phosphoinositide 3-kinase
PRMT6: Protein arginine methyltransferase 6
OVX: Ovariectomy
IGF: Insulin-like growth factor
m-ASCs: Mesenchymal adult stem cells
ASCs: Adipose derived stem cells
AMPK: Adenosine monophosphate-activated protein kinase
PTH: Parathyroid hormone
PTH1R: Parathyroid hormone 1 receptor
PPARγ: Peroxisome proliferator-activated receptor γ
ALP: Alkaline phosphatase
OPN: Osteopontin
FSH: Follicle-stimulating hormone
CREB: Cyclic-AMP response element-binding protein
CTSK: Cathepsin K
EGR1: Early growth response 1
CHI3L1: Chitinase 3-like protein 1
TLR4: Toll-like receptor 4
NFATc1: Nuclear factor of activated T-cells cytoplasmic 1
GPX4: Glutathione peroxidase 4
HuR: Human antigen R
PTPN6: Protein tyrosine phosphatase non-receptor type 6
RANKL: Receptor activator of NF-κB ligand
RANK: Receptor activator of NF-κB
TRAF6: Tumor necrosis factor receptor-associated factor 6
OPG: Osteoprotegerin
ZIC1: Zinc finger of the cerebellum 1
PHD2: Prolyl hydroxylase 2
VEGFA: vascular endothelial growth factor A
EPCs: Endothelial progenitor cells
VDAC3: Voltage-dependent anion channel 3
SNPs: Single nucleotide polymorphisms
GWAS: Genome-wide association studies
GEO: Gene Expression Omnibus
PBMCs: Peripheral blood mononuclear cells
